# Risk perception of the antimicrobial resistance by infection control specialists in Europe: a case-vignette study

**DOI:** 10.1186/s13756-020-0695-z

**Published:** 2020-02-14

**Authors:** Gabriel Birgand, Nico T. Mutters, Raheelah Ahmad, Evelina Tacconelli, Jean-Christophe Lucet, Alison Holmes, the Percept-R Study Group, Angel Asensio, Cagri Buke, Ljiljana Markovic-Denic, Maria da Graca Domingues Rocha, Jan Kluytmans, Outi Lyytikainen, Jonathan A. Otter, Elisabeth Presterl, Aidyn Salmanov, Kirsten Schaffer, Jesús Rodriguez Bano, Vered Schechner, Emese Szilagyi, Athanassios Tsakris

**Affiliations:** 1 NIHR Health Protection Research Unit in Healthcare Associated Infection and Antimicrobial Resistance at Imperial College London, Hammersmith Campus, Du Cane Road, London, W12 0NN UK; 2 European Committee on Infection Control (EUCIC), European Society of Clinical Microbiology and Infectious Diseases, Basel, Switzerland; 3 Medical Center – University of Freiburg, Institute for Infection Prevention and Hospital Epidemiology, Breisacher Straße 115B, 79106 Freiburg, Germany; 4 Division of Health Sciences, University of London, London, UK; 5 Department for Internal Medicine I, Division Infectious Diseases, Tübingen University, Otfried-Müller-Straße 10, 72076 Tübingen, Germany; 6 Department of Diagnostic and Public Health, Division Infectious Diseases, Verona University, Piazzale Luduvico Antonio Scuro 10, 37134 Verona, Italy; 7 Universite de Paris, Infection, Antimicrobiens, Modelisation, Evolution (IAME), Institut National de la Sante et de la Recherche Medicale (INSERM), Paris, France; 8 AP-HP, Hôpital Bichat – Claude Bernard, Infection Control Unit, F-75018 Paris, France

**Keywords:** Europe, Infection prevention and control, Antimicrobial resistance, Risk perception, Carbapenemase-producing *Enterobacteriaceae*, Meticillin-resistant *Staphylococcus aureus*, Carbapenemase-producing *Pseudomonas aeruginosa*, Carbapenemase-producing *Acinetobacter baumannii*, Vancomycin-resistant *Enterococci*

## Abstract

**Background:**

Using case-vignettes, we assessed the perception of European infection control (IC) specialists regarding the individual and collective risk associated with antimicrobial resistance (AMR) among inpatients.

**Methods:**

In this study, sixteen case-vignettes were developed to simulate hospitalised patient scenarios in the field of AMR and IC. A total of 245 IC specialists working in different hospitals from 15 European countries were contacted, among which 149 agreed to participate in the study. Using an online database, each participant scored five randomly-assigned case-vignettes, regarding the perceived risk associated with six different multidrug resistant organisms (MDRO). The intra-class correlation coefficient (ICC), varying from 0 (poor) to 1 (perfect), was used to assess the agreement for the risk on a 7-point Likert scale. High risk and low/neutral risk scorers were compared regarding their national, organisational and individual characteristics.

**Results:**

Between January and May 2017, 149 participants scored 655 case-vignettes. The perceptions of the individual (clinical outcome) and collective (spread) risks were consistently lower than other MDRO for extended spectrum beta-lactamase producing *Enterobacteriaceae* cases and higher for carbapenemase producing *Enterobacteriaceae* (CPE) cases. Regarding CPE cases, answers were influenced more by the resistance pattern (93%) than for other MDRO. The risk associated with vancomycin resistant *Enterococci* cases was considered higher for the collective impact than for the individual outcome (63% vs 40%). The intra-country agreement regarding the individual risk was globally poor varying from 0.00 (ICC: 0–0.25) to 0.51 (0.18–0.85). The overall agreement across countries was poor at 0.20 (0.07–0.33). IC specialists working in hospitals preserved from MDROs perceived a higher individual (local, *p* = 0.01; national, *p* < 0.01) and collective risk (local and national p < 0.01) than those frequently exposed to bacteraemia. Conversely, IC specialists working in hospitals with a high MDRO clinical burden had a decreased risk perception.

**Conclusions:**

The perception of the risk associated with AMR varied greatly across IC specialists and countries, relying on contextual factors including the epidemiology. IC specialists working in high prevalence areas may underestimate both the individual and collective risks, and might further negatively promote the MDRO spread. These finding highlight the need to shape local and national control strategies according to risk perceptions and contextual factors.

## Introduction

The burden of antimicrobial resistance (AMR) is highly heterogeneous across European countries [1]. The control of this phenomenon requires global and coordinated actions to prevent the spread of multidrug-resistant organisms (MDRO) [2]. Despite this urgent need, strategies for dealing with a common issue seem to be highly variable across neighbouring countries, regions and hospitals [3, 4]. In the same epidemiological context, some hospitals are implementing strong “search and isolate” strategies to control some MDROs, whereas others appear to take a more flexible approach [5].

The organisational framework, the socio-cultural and economic context seem to play a role in the differences of strategic approach. In some countries, infection control governance is centralised with a unique national recommendation, while others opt for a decentralised organisation with local adaptations [6]. Strategies may rely on variable organisations from coercive systems to governance based, the latter relies on the willingness of healthcare professionals to comply with best practices [7]. Furthermore, personal determinants, such as knowledge, belief, attitude and behaviours are closely linked to the cultural, local and organisational contexts in which decisions are made [8, 9]. We wanted to investigate if and how the perception of AMR by infection control (IC) specialists varies according to individual determinants (experience, position), the type of organisms, the local/national epidemiology, the organisational culture, the socio-cultural, and the economic context. Understanding the perceptions of IC specialists regarding MDRO and their associated risk to patients is a prerequisite for the coordinated implementation of interventions to prevent the emergence and dissemination of AMR in healthcare settings.

This case-vignette study aimed to assess the perception of the risk associated with MDRO by IC specialists across Europe in various clinical contexts. We also studied potential influencing factors at the national, organisational and individual levels.

## Methods

### Population and location of the study

This study involved IC specialists involved in the prevention of AMR in hospitals from 15 European countries (Austria, Finland, France, Greece, Germany, Hungary, Ireland, Israel, Netherland, Portugal, Serbia, Spain, Turkey, United Kingdom and Ukraine). The participants were recruited through national IC experts and members of the European Committee on Infection Control (EUCIC) from each country. These investigators were asked to recruit 16 IC specialists involved in AMR control, all working in different hospitals, leading to an expected number of 240 participants. Between March to June 2017, 245 IC experts were invited to participate in the study, and 149 agreed.

### Study design and data

We developed 16 case-vignettes based on real MDRO cases in hospitals, using a standard list of items. The case-vignettes were previously piloted and tested within the team of main investigators (GB, NTM, RA, ET, J-CL, AH). Each participant scored five randomly-assigned case-vignettes to allow assessments of the agreement across participants. For each vignette, the participants were questioned on their perception of the individual and collective risk associated with MDRO clinical cases according to a numerical 7-class Likert-type scale, ranging from “No risk” (score 1) to “High risk” (score 7) [10]. The individual risk was defined as the occurrence of a negative outcome for the colonised or infected patient. The collective risk was defined as the probability of MDRO acquisition for contact patients (i.e. by sharing the nursing staff with colonized patients). A secure online relational database was established for data collection. Before starting, each participant provided the following information: age, hospital category, and experience in the current position.

### Development of case-vignettes and questionnaires

We selected common AMR clinical cases experienced in hospitals across the participating countries. The following information were presented in vignettes: the epidemiological situation (sporadic vs epidemic), type of MDRO (three Methicillin-resistant *Staphylococcus aureus* (MRSA), four extended-spectrum beta-lactamase producing *Enterobacteriaceae* (ESBL-PE), four carbapenemase producing *Enterobacteriaceae* (CPE), two Carbapenemase-resistant *Acinetobacter baumannii* (CR-Ab), two vancomycin-resistant *Enterococci* (VRE), one Carbapenemase-resistant *Pseudomonas aeruginosa* (CR-Pa)), colonisation/infection, the timeline, the setting (type of ward, awareness of staff on IC measures, antibiotic use, workload, ward architecture and number of beds), and patients characteristics (colonisation/infection, autonomy, continence, devices, antibiotic treatments, repatriated). For each MDRO type, vignettes presented a various panel of epidemiological situations and patient’s characteristics in different settings. Cases details were presented chronologically in English language (Supplementary Figure S1 and Table S1).

### Individual, organisational and national factors

Participants completed a questionnaire developed to evaluate the AMR control strategy in their hospital. The 11 criteria selected for this form were informed by the IPC core components [11], including questions on the management (i.e. presence of IPC committee), resources, training programmes and AMR surveillance (Supplementary Table S2).

The local hospital epidemiology was assessed by the number of bacteraemia with the six selected MDROs observed in 2015 categorised in 0, 1–10 and > 10 episodes. Data on national epidemiology were extracted from the European Centre for Diseases prevention and Control (ECDC) surveillance atlas of Infectious diseases, providing the percentage of resistant isolates per species (resistant strains out of all strains) among invasive infections in 2015, and the Center for Disease Dynamics, Economics & Policy (CDDEP) resistance map 2016 for Turkey, and Serbia (Supplementary Table S3).

Individual cognitive factors for compliance with AMR control measures were assessed through a questionnaire (six questions) based on the “health belief model” [10]. This questionnaire enabled assessment of the individual decision and if levels of adherence with best practice is influenced by perceived susceptibility, perceived knowledge, intention to adhere (perceived practice), attitudes toward IC, perceived behavioural norms, perceived subjective norms, self-efficacy, and motivation (Supplementary Table S4).

The perception of the organisation and work conditions in hospitals were evaluated using four dimensions described in previous models [12, 13]. These models enabled assessment of teamwork (seven questions), management (nine questions), well-being and work conditions (seven questions), and stress and chaos in the work environment (two questions) at the hospital level (Supplementary Table S5). Items related to beliefs and perception were coded on a seven-point Likert scale, ranging from one “strongly disagree” to seven “strongly agree” with the statement of the item.

The socio-cultural factors included the national values for four dimensions of the Hofstede classification [14]: uncertainty avoidance (society’s tolerance for uncertainty and ambiguity), power distance (extent to which the less powerful members of organizations accept that power is distributed unequally), masculinity (masculine cultures based on ego, assertiveness and success; feminine on caring for the weak and quality of life), and individualism (degree to which people are integrated into groups). The socio-economic factors included Gross domestic product and the health expenditure per capita extracted from the Organisation for Economic Co-operation and Development (OECD) statistics 2013 [15] (Supplementary Table S6).

### Data analysis and statistics

#### Sample size calculation

We estimated the number of vignettes and participants needed to assess agreement based on the precision of the intraclass correlation coefficient (ICC) [16], with 16 vignettes each scored five times and an expected ICC of about 0.60, half the exact 95% confidence interval (95%CI). Each participant had to score five vignettes leading to an objective of 16 participants per country and 240 participants in total for the included 15 countries. Data were described as mean+/−SD, median (interquartile range), or percentage.

#### Data management and statistical analysis

The perceptions assessed through the seven-point Likert scale were dichotomised on one to five versus six and seven. A high-risk perception was attributed for six and seven versus low/neutral risk for the remaining five quotations. In the same way, items of the individual cognitive factors for compliance with AMR control measures, and the perception of the organisation and work conditions in hospitals were considered positive for six and seven (“agree and strongly agree”) versus negative for the remaining five quotations.

The analysis of factors associated with the risk perception was performed at the individual level. An aggregated score was calculated for each individual, organisational or national factor corresponding to the mean of quotations for each domain (aggregation of 11 criteria for the local IPC organisation, six criteria for the perception of individual IC compliance, seven criteria for teamwork, nine criteria for perception of management, seven criteria for stress and chaos, and two criteria for well-being and work conditions). Aggregated scores and values obtained for the individual, organisational or national factors were then dichotomized in high and low levels (<p75 or ≥ p75). The epidemiological indicators were categorised locally in three classes (0, 1- ≤ 10 or > 10 bacteraemia per year) and nationally following the seven ECDC classes of resistant isolates proportions.

Categorical variables were expressed as frequency (percentage). The ICC with the 95% confidence intervals (CI) were computed to evaluate agreements for the individual and collective perception based on 1–7 Likert scale scores. An ICC of zero indicates the level of agreement produced by chance alone and an ICC of one indicates perfect agreement. We defined poor agreement as ICC values less than 0.4, good agreement as ICC values of 0.4 to 0.7, and very good agreement as ICC values greater than 0.7 [17].

The individual, organisational and national factors were compared between the two groups of risk perception (low/neutral and high risk as defined above) using the Chi^2^ or their corresponding non-parametric versions, Fisher test, as appropriate. Analyses were performed using STATA, Version 12 (Stata Corp LP, College Station, TX).

### Ethics committee approval

Full ethical approval was obtained from the Health Research Authority (IRAS project ID is 192,130) and the Institutional Review Board (IRB 00006477) of HUPNVS, Paris 7 University, APHP before data collection. The completion of questionnaires inferred consent.

## Results

### Participants

A total of 149 IC specialists (62% out of 240 planned) from 15 European countries scored 655 case-vignettes (Table 1). Among them, 128 (86%) fully completed the questionnaire, and the remaining 21 participants quoted at least one case-vignette. The mean duration of practice in IC was 13.6 years (Median: 13 (8–20, min-max: 1–32), working in public general hospitals (*n* = 74, 50%), university hospitals (*n* = 72, 48%) and private hospitals (*n* = 3, 2%). These hospitals comprised an average of 791 acute care beds (Median: 641 beds) and 182 non-acute care beds (Median: 49 beds).

**Table 1:** Distribution of participants, case-vignettes quoted and questionnaires completed in each of the 15 European countries

Countries	Number of participants, N (%)	Number of case-vignettes quoted, N (%)
Austria	7 (5)	35 (5)
Finland	5 (3)	21 (3)
France	16 (11)	80 (12)
Germany	10 (7)	41 (6)
Greece	14 (9)	61 (9)
Hungary	10 (7)	42 (6)
Ireland	8 (5)	30 (5)
Israel	8 (5)	39 (6)
Netherlands	6 (4)	16 (2)
Portugal	7 (5)	18 (3)
Serbia	13 (9)	53 (8)
Spain	15 (10)	75 (11)
Turkey	10 (7)	50 (8)
UK	12 (8)	56 (9)
Ukraine	8 (5)	38 (6)
Total	149 (100)	655 (100)

### Risk perception

Overall, a high individual risk (clinical outcome) was perceived for 52% of the case-vignettes. This percentage varied according to countries (from 38% among Hungarian to 83% for Portuguese participants) and MDRO (from 29% (51 out of 173) for ESBL-PE cases to 73% (124/169) for CPE cases) (Table 2). Participants declared being influenced by the clinical situation in 85% of the 655 cases-vignettes quoted, by the resistance pattern of the organism in 81%, and by the type of organism (species) in 59% of cases (Supplementary Table S7). Regarding CPE cases, answers were influenced more by the resistance pattern (93%) than for other organisms. For CR-Pa, the clinical situation (93%), the resistance pattern (93%) and the type of organism (88%) were consistently, and highly influential on risk perception.

**Table 2:**
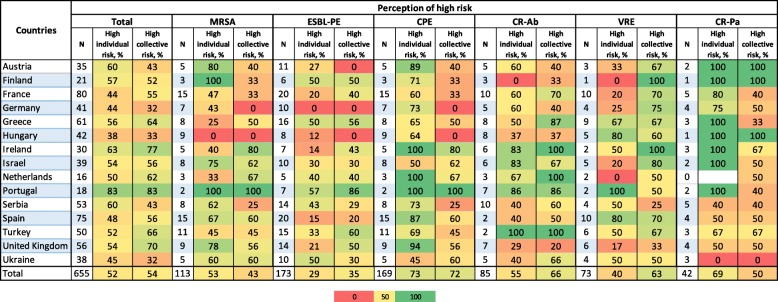
Proportion among the 149 European IC specialists perceiving a high individual and collective risk to patients globally and by type of multidrug-resistant organisms

Footnotes:

Individual risk corresponds to the risk of poor clinical outcomes for infected/colonised patients; collective risk corresponds to the risk for contact patients to become colonised and the transmission to other patients. N represents the total number of case-vignettes scored

Colours relate to the percentage of participants selecting the item where red = 0% of selection, yellow = 50% and green = 100% of selection by participants

*Abbreviations*: *IC* Infection control, *MRSA* Methicillin resistant *Staphylococcus aureus*, *ESBL-PE* Extended spectrum betalactamase producing *Enterobacteriaceae*, *CPE* Carbapenemase producing *Enterobacteriaceae*, *KPC Klebsiella pneumoniae* carbapenemase, *CR-Ab* Carbapenem-resistant *Acinetaobacter baumannii*, *VRE* Vancomycin resistant *Enterococci*, *CR-Pa* Carbapenemase Resistant-*Pseudomonas aeruginosa*

The collective risk was considered high in 54% of case-vignettes, ranging from 32% among German and Ukrainian to 83% for Portuguese participants. The perception of high collective risk ranged from 35% (61/173) for ESBL-PE cases to 72% (122/169) of quotations for CPE cases. The setting in which the clinical case was presented was the main influencing factor for the collective risk perception (82%) followed by clinical characteristics (69%), the resistance pattern (60%) and the type of organism (57%).

The risk associated with VRE cases was considered higher for the collective impact than for individual outcomes (63% vs 40%), whereas the individual risk was quoted higher than the collective risk for CR-Pa (69% vs 50%) and MRSA (53% vs 43%). The highest perceived collective and individual risks were associated with outbreaks due to CR-Ab and CPE, respectively, both occurring in ICU settings (data not shown).

**Fig. 1:**
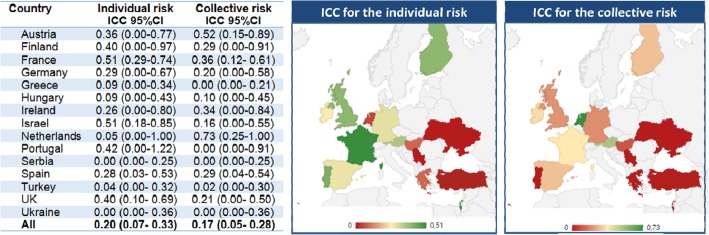
Overall assessment of agreement about risk perception within and across 15 European countries. Footnote: Individual risk corresponds to the risk of poor clinical outcomes for infected/colonised patients; collective risk corresponds to the risk for contact patients to become colonised and the transmission to other patients. N represents the total number of case-vignettes scored. The risk perception was scored on a 7-point Likert scale and then categorized in low risk for scores 1 to 3, neutral for a score of 4 and high risk for scores 5 to 7. Poor agreement: ICC < 0.4, Good agreement: ICC 0.4 to 0.7, Very good agreement: ICC > 0.7. Abbreviations: ICC, intraclass correlation; CI, confidence interval; UK, United Kingdom

For a same case-vignette, the agreement of respondents regarding the individual risk was low with an ICC of 0.20 (0.07–0.33). The interrater reliability of the collective risk was also low with an ICC of 0.17 (0.05–0.28) (Fig. 1). When looking at the agreement by country, the ICC of individual risk scores varied from 0.00 (95%CI, 0.00–0.25) for Serbia to 0.51 for France (0.29–0.74) and Israel (0.18–0.85). The ICC of collective risk scores varied from 0.00 (ICC: 0–0.21) in Greece to 0.73 (0.25–1) in the Netherlands.

### Relationship between risk perception and national, organisational and individual factors

A total of 128 IC specialists completed the questionnaires on the organisational and individual factors. In 2015, 58% of participants experienced > 10 ESBL-PE in their hospital and 48% > 10 MRSA bacteraemia. Regarding CPE and CR-Ab, 69% of participants identified less than 10 bacteraemia in their hospital (Supplementary Table S8). Finally, 42% of participants had not experienced VRE bacteraemia in 2015.

In median, participant’s hospitals were complying with 77% (IQR: 62–85%) of the 11 local IPC components. This percentage varied from 58% among Ukrainians to 88% for French hospitals. In median, 67% (IQR: 33–83%) of respondents declared complying with the six individual cognitive factors related to AMR control measures. This percentage varied from 50% (French, Irish, Portuguese, Turkish, British) to 100% for Finish participants (Supplementary Table S2 and Table S4).

The teamwork dimension was positive for 63% of IC specialists, 58% for the perception of management, 69% for well-being and work conditions and finally 53% for the dimension stress and chaos (Supplementary Table S5).

When looking at the relationships between the risk perception and contextual factors, the perception of a high individual and collective risk were both significantly correlated with a low local prevalence of MDRO bacteraemia (respectively *p* = 0.01 and 0.001) and a low national rate of resistant isolates among invasive infections (respectively *p* = 0.003 and < 0.001) (Table 3). The individual risk perception tended to be higher for IC specialists with less than five years of experience in the specialty.

**Table 3:** Factors associated with the risk perception to infected/colonised cases of antimicrobial resistance

Population-based variables	Individual risk			Collective risk		
	Number of participants with a Low/Neutral-risk perception^a^	Number of participants with a High-risk perception^a^	Univariate analysis (*p* value)	Number of participants with a Low/Neutral-risk perception^a^	Number of participants with a High-risk perception^a^	Univariate analysis (*p* value)
Participants years of practice as an IPC specialist (*n* = 128)						
< 5	3 (7)	13 (15)	0.10	5 (12)	11 (13)	1.00
≥ 5	48 (94)	64 (83)		38 (88)	874 (87)	
Type of healthcare facility of participants (*n* = 128)						
Private hospitals	1 (2)	1 (1)	0.86	0 (0)	2 (2)	0.34
Public general hospital	24 (47)	40 (52)		25 (58)	39 (46)	
University hospital	26 (51)	36 (47)		18 (42)	44 (52)	
Number of acute care beds (*n* = 128)						
< 300	13 (25)	16 (21)	0.72	10 (23)	19 (22)	0.97
300–600	11 (22)	21 (27)		10 (23)	22 (26)	
≥ 600	27 (53)	40 (52)		23 (54)	44 (52)	
Epidemiology of MDROs the year prior the study						
Local number of MDRO bacteremia in 2015^a^, (*n* = 461)						
0	42 (19)	64 (26)	**0.01**	15 (13)	91 (26)	**0.001**
1-≤ 10	81 (37)	107 (44)		45 (38)	143 (41)	
> 10	94 (43)	74 (30)		57 (49)	111 (32)	
National invasive infections, Resistant isolate % (*n* = 495)						
< 1%	33 (14)	60 (23)	**0.003**	10 (9)	83 (23)	< **0.001**
1-< 5%	24 (10)	38 (15)		7 (5)	55 (15)	
5-< 10%	13 (6)	25 (10)		11 (9)	27 (7)	
10-< 25%	59 (25)	41 (16)		39 (30)	61 (17)	
25-< 50%	32 (14)	35 (13)		21 (16)	46 (13)	
50-< 75%	14 (6)	6 (2)		5 (4)	15 (4)	
≥ 75%	59 (25)	56 (21)		35 (27)	80 (22)	
Local IPC organisation						
Low level	27 (53)	49 (64)	0.22	22 (51)	54 (64)	0.18
High level	24 (47)	28 (36)		21 (49)	31 (36)	
Individual cognitive factors for compliance with AMR control measures						
Low level	34 (67)	46 (60)	0.42	27 (63)	53 (62)	0.96
High level	17 (33)	31 (40)		16 (37)	32 (38)	
Perception of the organization and work conditions in participants hospital						
Teamwork						
Low level	43 (84)	56 (73)	0.12	34 (79)	65 (76)	0.74
High level	8 (16)	21 (27)		9 (21)	20 (24)	
Perception of management						
Low level	42 (82)	54 (70)	0.12	33 (77)	63 (74)	0.75
High level	9 (18)	23 (30)		10 (23)	22 (26)	
Stress and chaos						
Low level	41 (80)	60 (78)	0.74	37 (86)	64 (75)	0.16
High level	10 (20)	17 (22)		6 (14)	21 (25)	
Well-being and work conditions						
Low level	37 (73)	53 (69)	0.65	31 (72)	59 (69)	0.75
High level	14 (27)	24 (31)		12 (28)	26 (31)	
National socio-cultural factors						
Power Distance						
Low level	36 (71)	59 (77)	0.44	28 (65)	67 (79)	0.09
High level	15 (29)	18 (23)		15 (35)	18 (21)	
Uncertainty Avoidance						
Low level	41 (80)	55 (71)	0.25	32 (74)	64 (75)	0.91
High level	10 (20)	22 (29)		11 (26)	21 (25)	
Individualism						
Low level	33 (65)	57 (74)	0.26	28 (65)	62 (73)	0.36
High level	18 (35)	20 (26)		15 (35)	23 (27)	
Masculinity						
Low level	34 (67)	54 (70)	0.68	24 (60)	64 (75)	0.02
High level	17 (33)	23 (30)		19 (40)	21 (25)	
Socio-economic factors						
GDP per capita						
Low level	41 (80)	63 (82)	0.84	33 (80)	71 (84)	0.35
High level	10 (20)	14 (18)		10 (20)	14 (16)	
Health expenditure per capita						
Low level	27 (53)	50 (65)	0.17	18 (42)	59 (69)	**0.003**
High level	24 (47)	27 (35)		25 (58)	26 (31)	

Footnotes:

Individual risk corresponds to the risk of poor clinical outcomes for infected/colonised patients; collective risk corresponds to the risk for contact patients to become colonised and the transmission to other patients

^a^Low/Neutral-risk perception: Mean Scores = 1–5; High-risk perception: Mean Scores = 6–7

Power distance index (PDI): The power distance index is defined as “the extent to which the less powerful members of organizations and institutions accept and expect that power is distributed unequally”. A higher degree of the Index indicates that hierarchy is clearly established and executed in society, without doubt or reason. A lower degree of the Index signifies that people question authority and attempt to distribute power

Uncertainty avoidance (UAI): The uncertainty avoidance index is defined as “a society’s tolerance for ambiguity”, in which people embrace or avert an event of something unexpected, unknown, or away from the status quo. Societies that score a high degree in this index opt for stiff codes of behaviour, guidelines, laws, and generally rely on absolute truth, or the belief that one lone truth dictates everything and people know what it is. A lower degree in this index shows more acceptance of differing thoughts or ideas

Individualism vs. collectivism (IDV): This index explores the “degree to which people in a society are integrated into groups”. Individualistic societies have loose ties that often only relate an individual to his/her immediate family. They emphasize the “I” versus the “we”. Its counterpart, collectivism, describes a society in which tightly-integrated relationships tie extended families and others into in-groups. These in-groups are laced with undoubted loyalty and support each other when a conflict arises with another in-group

Masculinity vs. femininity (MAS): In this dimension, masculinity is defined as “a preference in society for achievement, heroism, assertiveness and material rewards for success”. In feminine societies, they share modest and caring views equally with men. In more masculine societies, women are somewhat assertive and competitive, but notably less than men. In other words, they still recognize a gap between male and female values

*Abbreviations*: *MDRO* Multi-drug resistant organisms, *IPC* Infection prevention and control

The risk perception tended to be lower among participants working in hospitals with a high level of IPC organisation, embedded in national cultures based on masculinity, individualism, power distance, and in countries with a high degree of gross domestic product and health expenditure. On the other hand, a high-risk perception was globally observed among participants selecting compliance with AMR control measures, and based in hospitals with a high level of work conditions.

## Discussion

Agreement regarding the risk perception of AMR by IC specialists varied greatly across countries, individuals and MDRO. The risk was consistently perceived low for ESBL-PE cases and high for CPE cases. IC specialists working in an environment preserved from MDRO were characterised by their high perception of the individual and collective risk. Conversely, IC specialists frequently confronted with MDRO cases had a reduced risk perception, potentially due to habituation. Individual positive beliefs on work conditions and compliance with best practices were more prevalent among participants with a high-risk perception.

Nationally, IC specialists perceived CPE and CR-Pa as of higher individual importance, and VRE or CR-Ab as a collective issue. CPE and CR-Pa infections are widely recognised to be associated with poor clinical outcomes, especially among ICU patients [18, 19]. On the other hand, VRE and CR-Ab are known to have a strong potential for spreading [20]. In 2015 in Europe, third generation cephalosporin-resistant *Enterobacteriaceae* represented the largest part of the burden caused by antibiotic-resistant bacteria [1]. However, carbapenemase-resistant *K. pneumoniae* showed the most significant positive trend in attributable deaths between 2007 and 2015. The results on risk perception found for ESBL-PE in the present study are not in line with this estimated burden. A jump of public health attention from MRSA in the 2000s to CPE in the 2010s, due to the rapid evolution of AMR among *Enterobacteriaceae,* may have led to a rapid shift of attentiveness explaining this result. In a context of rising resistance levels, we may assume that resources are devoted to the control of the MDRO considered the most risky. According to our results, efforts may focus on the control of CPE and CR-Pa mainly due to their resistance pattern.

The local and national epidemiology of MDRO invasive infections were the main factors associated with a high-risk perception. The high level of risk perception by IC specialists may come from an external pressure formalised by a strong policy and commitment for the control of MDRO. In this context, hospitals are enforced to strictly implement control measures, leading to a low AMR burden. Conversely, IC specialists working in hospitals with a low incidence of MDRO may perceive each emerging situation as a risk to patients. In reaction, they would probably apply strict measures following national recommendations if available. In hospitals with a high AMR burden, the experience accumulated on MDRO, and resources necessary for their control, may both alleviate the risk perception. In endemic situations, as for ESBL-PE in many European countries, controlling the spread of MDRO appears complex and requiring large resources. In this case, hospitals and IC specialists may not strictly apply guidelines, and operate in downgraded mode softening the strategy. IC specialists frequently managing MDRO outbreaks may get into a routine, with a lighter vision of the risk.

IC specialists’ perceiving high risks were also those with a consistent low (but non-significant) level of local IPC organisation, national socio-cultural (based on femininity, collectivism and a low power distance), and/or socio-economic factors. This same group selected compliance with AMR control measures, and thought their hospital to have a fair to good level of work conditions. In this context, the level of risk perception may rely on the lack of IPC infrastructure and resources, in organisations without clear leadership. Defining clear priorities and engaging key stakeholders on the strategy for the control of MDRO at the organisational level constitute the first perspectives of improvement. In diametrically opposed contexts, of low-level perception of risks, actions should focus on raising individual IPC specialist capability to comply with control measures, and emphasising the horizontal approach of AMR prevention with more collective decision-making, integrated and team-based initiatives.

Several limitations need to be acknowledged. First, the vignettes were scored for the risk perception and the IC strategy by each participant in isolation which is contra to most working practices. MDRO situations are often complex, typically requiring discussion and collegial decision making to adapt the strategy to the current context (clinical, setting, and resources). Second, the vignettes were scored via an online database. The vignettes were built from real cases, but the perception and decision making may be different than in the reality. Moreover, despite definitions provided for each criteria, the variability of answers may be explained by the variability of reading and understanding of case-vignettes presented in English language. Third, in some countries we did not reach the 16 expected IC specialists for participation. Finally, participants in each country were solicited by national leaders in the field of AMR surveillance and prevention. This recruitment method potentially induced selection bias with a majority of participants working in universities or large hospitals. However, IC specialists working in these centres presumably have the most experience with MDRO. According to these, we may expect getting reliable answers on the risk perception, reinforcing the theoretical role of individual and contextual determinants.

## Conclusion

The risk perception regarding similar MDRO cases varied across IC specialists and countries, relying on contextual factors, including the epidemiology. A shared perception of the risks represents an important prerequisite to the harmonization of actions. For IC specialists with low risk perception, the prominence of accountability and empowerment in the management of AMR seem to be key elements of AMR control strategies.

## Supplementary information

**Additional file 1: Figure S1.** Example of case-vignette. **Table S1.** Detailed description of case-vignette scenarios developed for the survey. **Table S2.** Organisation, management, and structure for the control of antimicrobial resistance in participants hospitals. **Table S3.** Percentage of resistant isolates per species (resistant strains out of all strains) from invasive infections in 2015, European Centre for Diseases prevention and Control (ECDC) surveillance atlas of Infectious diseases, and CDDEP resistance map (Turkey, and Serbia in 2016). **Table S4.** Individual cognitive factors for compliance with antimicrobial resistance control measures (*n* = 128 participants). **Table S5.** Perception of the organisation and work conditions in your hospital. **Table S6.** Socio-cultural and socio-economic factors. **Table S7.** Factors influencing participants in their quotation of the individual and collective risk. **Table S8.** Epidemiology of MDROs in your hospital in 2015.

## Data Availability

The datasets generated and analysed during the current study are not publicly available due to confidentiality clauses but anonymised versions are available from the corresponding author on reasonable request.
