# Supplementary material for: Risk perception of the antimicrobial resistance by infection control specialists in Europe: a case-vignette study
Source: Antimicrob Resist Infect Control. 2020 Feb 14;9:33. doi: 10.1186/s13756-020-0695-z (PMC7023755; doi:10.1186/s13756-020-0695-z)
Supplement: Supplementary file 1 — Additional file 1: Figure S1. Example of case-vignette. Table S1. Detailed description of case-vignette scenarios developed for the survey. Table S2. Organisation, management, and structure for the control of antimicrobial resistance in participants hospitals. Table S3. Percentage of resistant isolates per species (resistant strains out of all strains) from invasive infections in 2015, European Centre for Diseases prevention and Control (ECDC) surveillance atlas of Infectious diseases, and CDDEP resistance map (Turkey, and Serbia in 2016). Table S4. Individual cognitive factors for compliance with antimicrobial resistance control measures (n = 128 participants). Table S5. Perception of the organisation and work conditions in your hospital. Table S6. Socio-cultural and socio-economic factors. Table S7. Factors influencing participants in their quotation of the individual and collective risk. Table S8. Epidemiology of MDROs in your hospital in 2015. [file 13756_2020_695_MOESM1_ESM.doc]

**Figure S1. Example of case-vignette.**

**Scenario 10: Carbapenemase producing *Enterobacteriaceae***

**Alert**

- You are arriving in a hospital as the new IPC manager.
- The position was vacant during the previous year before your arrival.
- During a meeting with the head of microbiology, they are explaining that several CPE have been cultures the past 2 months

**Epidemic description**

- 19 cases of CPE Enterobacter cloacae OXA-48 cultured from various types of specimens
  - 2 cases of CLABSI among which one bacteriemia
  - 3 cases of bronchial aspirations
  - 6 urines
  - 8 faecal samples or rectal swabs
- All strains have the same phenotype
- 12 different sectors of your hospital have been frequented by these patients
- No particular precautions have been undertaken

**Epidemic curve**


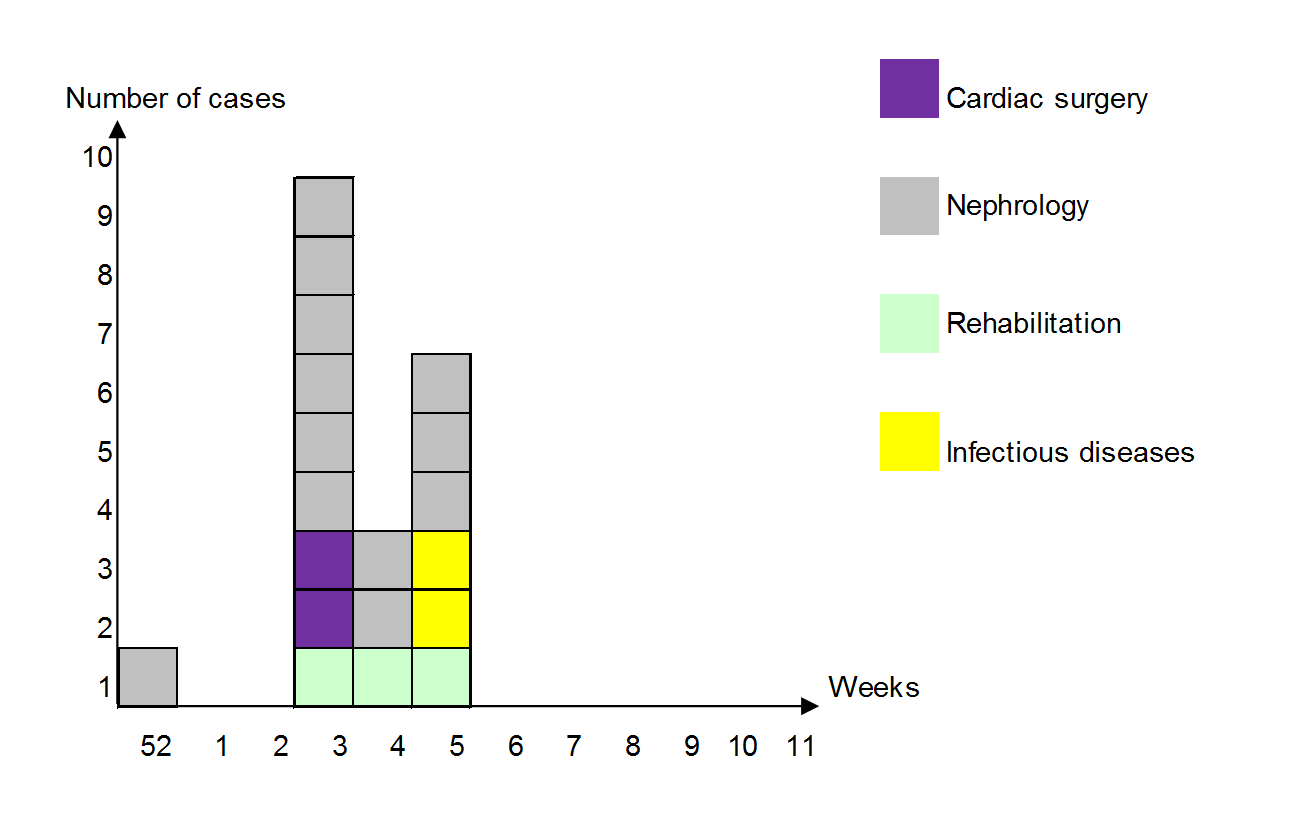


**Synoptic table**


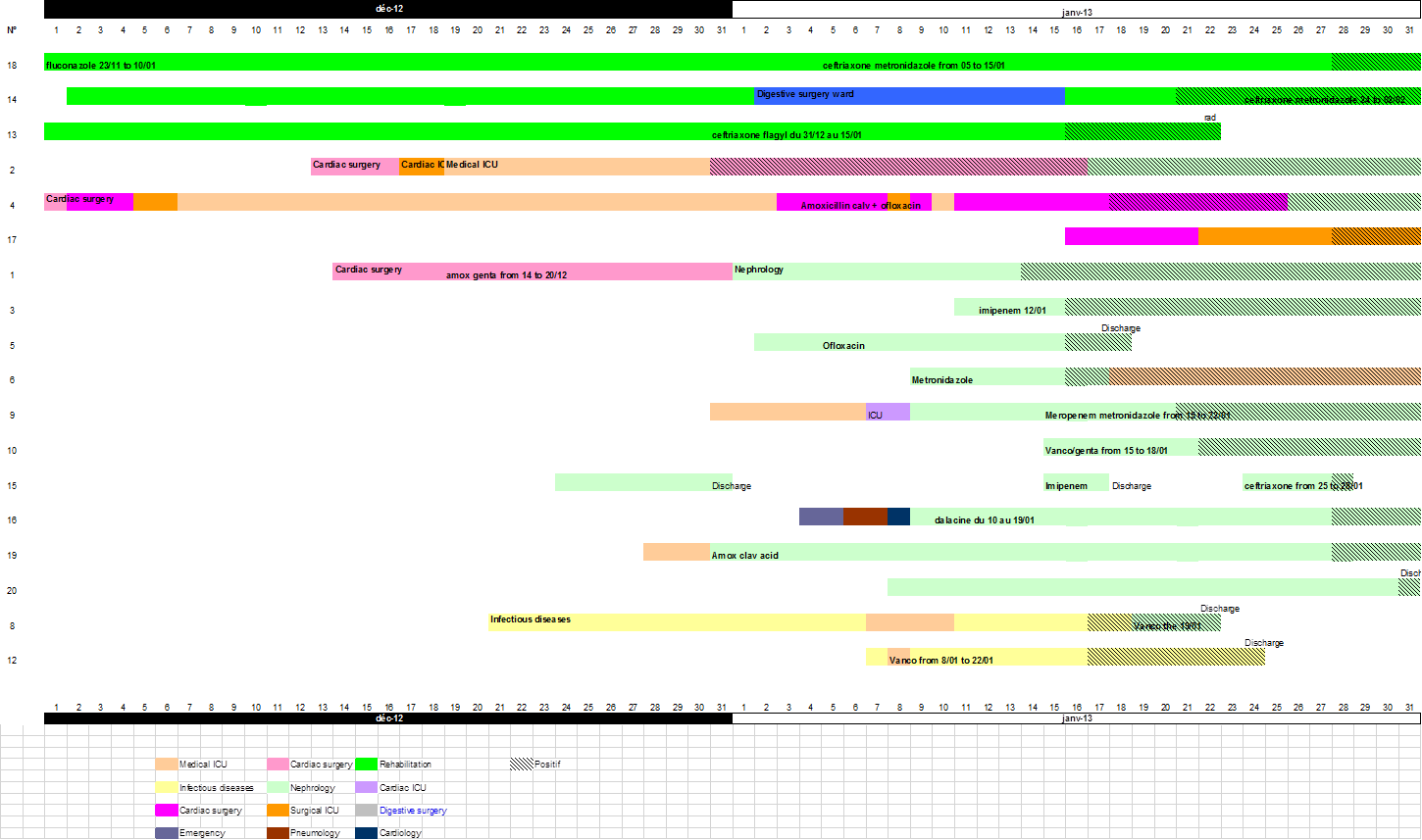


**Setting**

- Ward speciality: Cardiac, ICUs, Nephrology, rehabilitation, ID
- Size of the hospital: 400 beds
- Awareness of the unit: Low Infection Control culture
- Leadership: Low from the administrative and medical part
- Antibiotic consumption: High in Nephrology, ID, ICU. Low in others
- Level of Hand hygiene: Low with 30% of compliance in the hospital
- Workload: Intermediate
- Number of patients sharing the nursing staff with CPE patient: Approximately 450 patients
- Architecture: 5% of single rooms in the hospital. Open space in ICU, multiple bedroom in medical and surgical wards

**Table S1. Detailed description of case-vignette scenarios developed for the survey.**


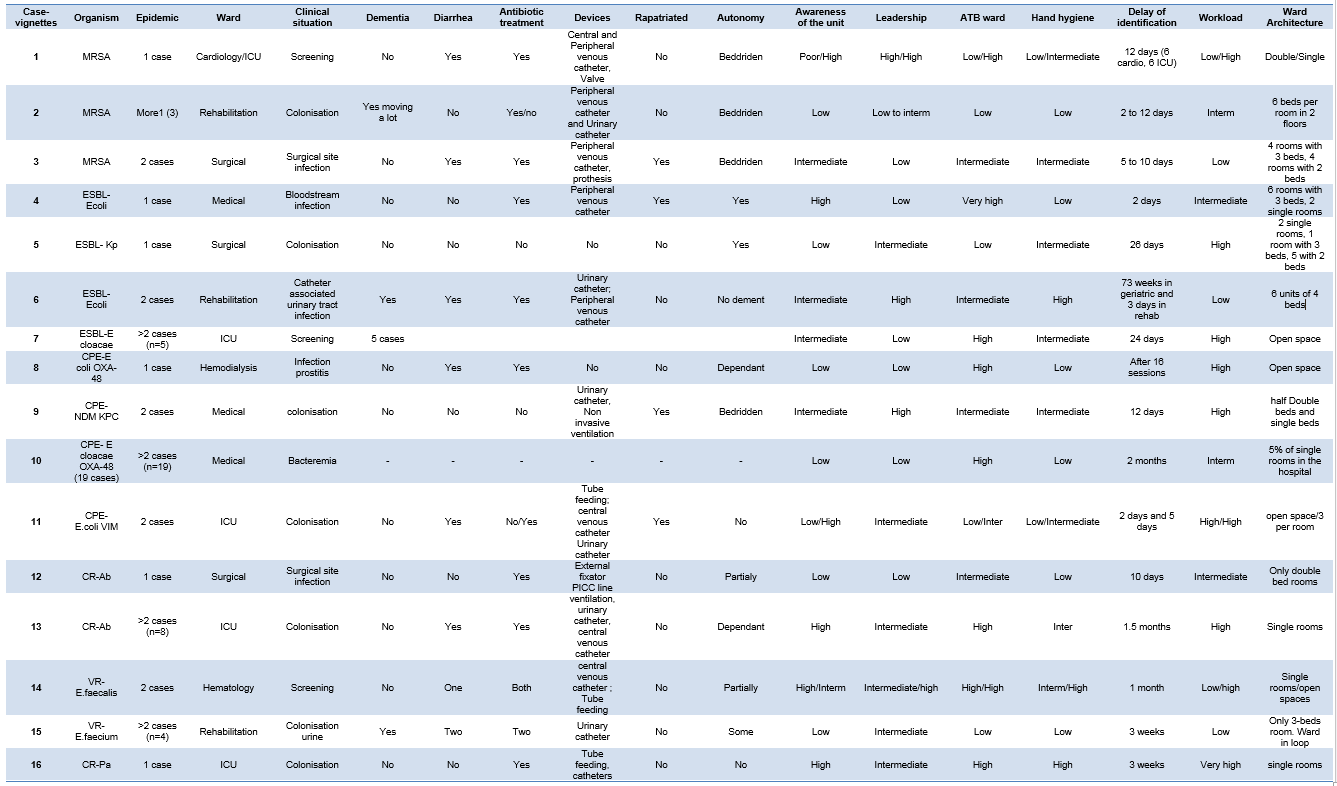


Abbreviations: ICU, intensive care unit; MRSA, methicillin resistant *Staphylococcus aureus*; ESBLPE, extended spectrum betalactamase producing *Enterobacteriaceae*; CPE, carbapenemase producing *Enterobacteriaceae*; KPC, *Klebsielle pneumoniae* carbapenemase; CR-Ab, carbapenem resistant *Acinetaobacter baumannii*; Ab, *Acinetaobacter baumannii*; VRE, vancomycin resistant *Enterococci*; CR-*Pa;* Carbapenemase Resistant-*Pseudomonas aeruginosa.*

Footnote:

Awareness of the unit: level of knowledge in the team regarding the type of multi-drug resistant organism, the way of transmission and control measures, mainly link to their experience dealing with this situation;

Leadership: level of leadership in the unit by managers of the unit (medical head and lead nurse), leadership in general and especially in the field of infection control measures (presence in the ward, recognition by team members...) Efficiency of the management in transmitting best practices;

Level of hand hygiene: Compliance rate according to the 5 moments of the WHO;

Workload: Factor reflecting the level of bed occupancy in the unit, the staffing and workload in general;

Architecture: This variable gives some informations on the configuration of the unit and the proximity of patients hospitalised in the unit (mainly based on the availability of single room)

**Table S2. Organisation, management, and structure for the control of antimicrobial resistance in participants hospitals.**

| **Characteristics** | **n/N=128 (%)** |
| --- | --- |
| 1. Is an Infection Prevention and Control committee in place within your hospital? | 124 (97) |
| 2. Is infection control included on the hospital administration agenda? | 116 (91) |
| 3. Are there defined goals to prevent antimicrobial resistance? (e.g. antimicrobial resistance rates) | 88 (69) |
| 4. Is the staffing for infection control appropriate? | 97 (76) |
| 5. In your opinion, is the microbiology laboratory accurate in identifying multidrug resistant organisms? | 106 (82) |
| 6. Is alcohol-based hand rub available at the point of care? | 126 (98) |
| 7. Are sinks stocked with soap and single-use towels? | 111 (87) |
| 8. Are national/international guidelines for antimicrobial resistance control adapted to your local situation? | 106 (83) |
| 9. What is the approximate average percentage of single bed rooms in your hospital? (median, IQR) | 15 (5-22) |
| 10. Is there a teaching programme based on local antimicrobial resistance prevention guidelines in your hospital? | 80 (62) |
| 11. Are you participating in (inter-) national antimicrobial resistance surveillance initiatives? | 102 (80) |

**Table S3. Percentage of resistant isolates per species (resistant strains out of all strains) from invasive infections in 2015, European Centre for Diseases prevention and Control (ECDC) surveillance atlas of Infectious diseases, and CDDEP resistance map (Turkey, and Serbia in 2016).**

|  | **MRSA** | **3CG-R *E.c*** | **3CG-R *K.p*** | **CR-*E.c*** | **CR- *K.p*** | **CR-Ab** | **VR-*E.faecium*** | **VR-*E.faecalis*** | **CR-Pa** |
| --- | --- | --- | --- | --- | --- | --- | --- | --- | --- |
| 1: Austria | 7.5 | 9.5 | 8.4 | 0.8 | 0 | 9.4 | 3.1 | 0 | 12.2 |
| 3: Finland | 1.9 | 6.1 | 3 | 0 | 0 | 2.3 | 0.3 | 0 | 4.7 |
| 4: France | 15.7 | 11 | 30.5 | 0.5 | 0 | 5.6 | 0.8 | 0.3 | 16.4 |
| 5: Germany | 11.2 | 10.3 | 10.2 | 0.1 | 0 | 6.5 | 10.5 | 0.2 | 14.7 |
| 6: Greece | 39.4 | 19.8 | 69.5 | 61.9 | 1.2 | 93.5 | 19.7 | 1.4 | 40.4 |
| 7: Hungary | 24.7 | 16.7 | 37.2 | 0.1 | 0 | 55.2 | 16.7 | 0 | 35.8 |
| 8: Ireland | 18.1 | 11.4 | 14.7 | 0.5 | 0 | 6 | 45.8 | 1.4 | 9.2 |
| 9: Israel | - | - | - | - | - | - | - | - | - |
| 11: Netherlands | 1.3 | 5.7 | 8.6 | 0.1 | 0 | 4.1 | 1.4 | 0 | 4.0 |
| 13: Portugal | 46.8 | 16.1 | 40.4 | 3.4 | 0.1 | 57.7 | 20.3 | 2.0 | 19.8 |
| 14: Serbia | 42 | 35 | 90 | 2 | 40 | 97 | 35 | 9 | 48 |
| 15: Spain | 25.3 | 11.6 | 20.3 | 2.2 | 0 | 53.7 | 2.5 | 0.1 | 22.7 |
| 18: Turkey | 26 | 52 | 56 | 11 | 5 | 93 | 15 | 1 | 24 |
| 19: UK | 10.8 | 11.3 | 10.5 | 0.4 | 0.3 | 0.8 | 17 | 3.6 | 2.4 |
| 20: Ukraine | - | - | - | - | - | - | - | - | - |

Abbreviations: MRSA, methicillin resistant *Staphylococcus aureus*; ESBLPE, extended spectrum betalactamase producing *Enterobacteriaceae*; CPE, carbapenemase producing *Enterobacteriaceae*; KPC, *Klebsielle pneumoniae* carbapenemase; CR-Ab, carbapenem resistant *Acinetaobacter baumannii*; Ab, *Acinetaobacter baumannii*; VRE, vancomycin resistant *Enterococci*; CR-*Pa;* Carbapenemase Resistant-*Pseudomonas aeruginosa*

**Table S4. Individual cognitive factors for compliance with antimicrobial resistance control measures (n=128 participants)**

| **Characteristics** | **Quotations >5 (n,%)**  Strongly agree |
| --- | --- |
| 1. I am perfectly aware of recommendations regarding best practices for the prevention of antimicrobial resistance | 94 (73) |
| 2. I think strict adherence to national guidelines for antimicrobial resistance control is necessary | 94 (73) |
| 3. My colleagues in other hospitals respect national recommendations for the control of antimicrobial resistance | 40 (31) |
| 4. The way I follow national recommendations for antimicrobial resistance control is a good example to follow for my colleagues in other hospitals | 69 (54) |
| 5. I feel able to apply national guidelines for antimicrobial resistance control in my hospital | 64 (50) |
| 6. I am open to improve my practices in antimicrobial resistance prevention | 109 (85) |

**Table S5. Perception of the organisation and work conditions in your hospital.**

| **Characteristics** | **Quotations >5 (n,%)**  **Strongly agree** |
| --- | --- |
| **Dimension 1: Teamwork** |  |
| 1. In general, when there is conflict in the hospital, people talk about it and solve any issues successfully | 63 (50) |
| 2. There is tension within teams (in general) in this hospital | 49 (39) |
| 3. I am optimistic about the capacity of the hospital to ensure good relationships between professionals | 82 (65) |
| 4. The infection control team has worked to build relationships between members of the hospital | 98 (77) |
| 5. The hospital members work together | 83 (65) |
| 6. The success of teams are due in major part to team work | 104 (82) |
| 7. I am optimistic about for the capacity of teams to prevent antimicrobial resistance | 82 (65) |
| **Dimension 2: Perception of management** |  |
| 1. My suggestions about the control of antimicrobial resistance would be acted upon if I expressed them to ward managers | 90 (71) |
| 2. The chief executive board is available to deal with problems | 68 (54) |
| 3. The chief executive board help us to create our own solutions to improve the quality of care | 73 (57) |
| 4. Ideas coming from the infection control team in this hospital have been implemented to improve antimicrobial resistance prevention | 95 (75) |
| 5. Front-line staff in the hospital are involved in the implementation of measures to improve antimicrobial resistance prevention | 83 (65) |
| 6. Managers of the hospital/wards encourage team members to take initiatives | 68 (54) |
| 7. Decisions in this hospital are taken by the management with a poor involvement of front-line staff | 53 (42) |
| 8. I am optimistic about the capacity of teams to take the initiative to improve patient care | 81 (64) |
| 9. Clinical team members are motivated to improve antibiotic prescribing | 60 (47) |
| **Dimension 3: Stress and chaos** |  |
| 1. It is difficult to change things in this hospital because of the workload related to patient care | 81 (64) |
| 2. Professionals have difficulties remembering all infection control measures for a daily implementation | 93 (73) |
| 3. I feel stressed in this hospital | 44 (35) |
| 4. This hospital is badly organised | 34 (27) |
| 5. The hospital is not open to changes to improve patient care | 28 (22) |
| 6. The teams feel overwhelmed by the workload | 98 (77) |
| 7. Things are going so fast in this hospital that it is difficult to know what is happening | 35 (28) |
| **Dimension 4: Well-being and work conditions** |  |
| 1. When a protocol/recommendation appears difficult to me, I still feel able to make good efforts to implement it | 93 (73) |
| 2. I feel able to apply infection control recommendations even if they go against the opinions of my colleagues | 81 (64) |

**Table S6. Socio-cultural and socio-economic factors.**

|  | **Power Distance (PDI)** | **Uncertainty Avoidance (UAI)** | **Long Term Orientation vs. Short Term Normative Orientation (LTO)** | **GDP per capita, $** | **Health expenditure per capita, $** |
| --- | --- | --- | --- | --- | --- |
| 1: Austria | 11 | 70 | 60 | 48,000 | 4,553 |
| 3: Finland | 33 | 59 | 38 | 42,200 | 3,442 |
| 4: France | 68 | 86 | 63 | 42,300 | 4,124 |
| 5: Germany | 35 | 65 | 83 | 48,100 | 4,819 |
| 6: Greece | 60 | 100 | 45 | 26,700 | 2,366 |
| 7: Hungary | 46 | 82 | 58 | 27,500 | 1,719 |
| 8: Ireland | 28 | 35 | 24 | 69,200 | 3,663 |
| 9: Israel | 13 | 81 | 38 | 35,200 | 2,426 |
| 11: Netherlands | 38 | 53 | 67 | 51,000 | 5,131 |
| 13: Portugal | 63 | 99 | 28 | 28,900 | 2,514 |
| 14: Serbia | 86 | 92 | 52 | 14,500 | - |
| 15: Spain | 57 | 86 | 48 | 36,400 | 2,898 |
| 18: Turkey | 66 | 85 | 46 | 24,900 | 941 |
| 19: UK | 35 | 35 | 51 | 8,300 | 3,235 |
| 20: Ukraine | 92 | 95 | 55 | 42,500 | - |

Footnotes:

Power distance index (PDI): The power distance index is defined as "the extent to which the less powerful members of organizations and institutions accept and expect that power is distributed unequally". A higher degree of the Index indicates that hierarchy is clearly established and executed in society, without doubt or reason. A lower degree of the Index signifies that people question authority and attempt to distribute power

Uncertainty avoidance (UAI): The uncertainty avoidance index is defined as "a society's tolerance for ambiguity", in which people embrace or avert an event of something unexpected, unknown, or away from the status quo. Societies that score a high degree in this index opt for stiff codes of behaviour, guidelines, laws, and generally rely on absolute truth, or the belief that one lone truth dictates everything and people know what it is. A lower degree in this index shows more acceptance of differing thoughts or ideas.

Long-term orientation vs. short-term orientation (LTO): This dimension associates the connection of the past with the current and future actions/challenges. A lower degree of this index (short-term) indicates that traditions are honored and kept, while steadfastness is valued. Societies with a high degree in this index (long-term) view adaptation and circumstantial, pragmatic problem-solving as a necessity.

Masculinity vs. femininity (MAS): In this dimension, masculinity is defined as "a preference in society for achievement, heroism, assertiveness and material rewards for success". In feminine societies, they share modest and caring views equally with men. In more masculine societies, women are somewhat assertive and competitive, but notably less than men. In other words, they still recognize a gap between male and female values.

**Table S7. Factors influencing participants in their quotation of the individual and collective risk.**

| **What is influencing you for this answer?** | **The type of organism (species) N (%)** | **The resistance pattern**  **N (%)** | **The clinical situation**  **N (%)** | **The setting N (%)** |
| --- | --- | --- | --- | --- |
| MRSA positive patients | 71 (62.8) | 80 (70.8) | 107 (94.7) |  |
| MRSA contact patients | 75 (66.4) | 59 (52.2) | 73 (64.6) | 99 (87.6) |
| ESBLPE positive patients | 85 (49.1) | 100 (57.8) | 144 (83.2) |  |
| ESBLPE contact patients | 83 (48.0) | 103 (59.5) | 123 (71.1) | 139 (80.3) |
| CPE positive patients | 91 (53.8) | 157 (92.9) | 139 (82.2) |  |
| CPE contact patients | 89 (52.7) | 117 (69.2) | 115 (68.0) | 142 (84.0) |
| CR-Ab positive patients | 61 (71.8) | 67 (78.8) | 69 (81.2) |  |
| CR-Ab contact patients | 58 (68.2) | 53 (62.4) | 63 (74.1) | 65 (76.5) |
| VRE positive patients | 43 (58.9) | 52 (71.2) | 57 (78.1) |  |
| VRE contact patients | 40 (54.8) | 37 (50.7) | 51 (69.9) | 61 (83.6) |
| CR-*Pa* positive patients | 37 (88.1) | 39 (92.9) | 39 (92.9) |  |
| CR-*Pa* contact patients | 26 (61.9) | 25 (59.5) | 29 (69.0) | 28 (66.7) |

Abbreviations: MRSA, methicillin resistant *Staphylococcus aureus*; ESBLPE, extended spectrum betalactamase producing *Enterobacteriaceae*; CPE, carbapenemase producing *Enterobacteriaceae*; KPC, *Klebsielle pneumoniae* carbapenemase; CR-Ab, carbapenem resistant *Acinetaobacter baumannii*; Ab, *Acinetaobacter baumannii*; VRE, vancomycin resistant *Enterococci*; CR-*Pa;* Carbapenemase Resistant-*Pseudomonas aeruginosa*

**Table S8. Epidemiology of MDROs in your hospital in 2015.**

| **Countries** | **Overall**  **N= 128** |
| --- | --- |
| **15. ESBL-PE** |  |
| 0 episode per year | 1 (1) |
| 1 to 10 episodes per year | 42 (32) |
| >10 episodes per year | 75 (58) |
| Unknown | 10 (8) |
| **16. CPE** |  |
| 0 episode per year | 42 (32) |
| 1 to 10 episodes per year | 48 (37) |
| >10 episodes per year | 30 (23) |
| Unknown | 8 (6) |
| **17. Carbapenems resistant- *Pseudomonas aeruginosa*** |  |
| 0 episode per year | 22 (17) |
| 1 to 10 episodes per year | 58 (45) |
| >10 episodes per year | 36 (28) |
| Unknown | 12 (9) |
| **18. Carbapenems resistant *Acinetobacter baumannii*** |  |
| 0 episode per year | 46 (35) |
| 1 to 10 episodes per year | 40 (31) |
| >10 episodes per year | 33 (25) |
| Unknown | 9 (7) |
| **19. MRSA** |  |
| 0 episode per year | 9 (7) |
| 1 to 10 episodes per year | 62 (48) |
| >10 episodes per year | 49 (38) |
| Unknown | 8 (6) |
| **20. Glycopeptide resistant *E.faecium*** |  |
| 0 episode per year | 54 (42) |
| 1 to 10 episodes per year | 39 (30) |
| >10 episodes per year | 21 (16) |
| Unknown | 14 (10) |

Abbreviations: MRSA, methicillin resistant *Staphylococcus aureus*; ESBLPE, extended spectrum betalactamase producing *Enterobacteriaceae*; CPE, carbapenemase producing *Enterobacteriaceae*; KPC, *Klebsielle pneumoniae* carbapenemase; CR-Ab, carbapenem resistant *Acinetaobacter baumannii*; Ab, *Acinetaobacter baumannii*; VRE, vancomycin resistant *Enterococci*; CR-*Pa;* Carbapenemase Resistant-*Pseudomonas aeruginosa*
